# COVID-19 and the second exams fiasco across the UK: four nations trying to avoid immediate policy failure

**DOI:** 10.1057/s41293-022-00202-1

**Published:** 2022-03-23

**Authors:** Sean Kippin, Paul Cairney

**Affiliations:** University of Stirling, Stirling, FK9 4LA UK

**Keywords:** COVID-19, Education policy, UK and devolved government, Multiple streams analysis, Policy learning and transfer, Crisis management, Policy success

## Abstract

In 2021, the UK and devolved governments tried to avoid the school exams fiasco of 2020. Their immediate marker of success was to prevent a similar U-turn on their COVID-19 school exams replacement policies. They still cancelled the traditional exam format, and sought teacher assessments to determine their grades, but this time without using an algorithm to standardise the results. The outcomes produced *some* concerns about inequity, since the unequal exam results are similar to those experienced in 2020. However, we did not witness the same sense of acute political crisis. We explain these developments by explaining this year’s ‘windows of opportunity’ overseen by four separate governments, in which the definition of the problem, feasibility of each solution, and motive of policymakers to select one, connects strongly to the previous U-turn. A policy solution that had been rejected during the first window became a lifeline during the second and a likely choice during the third. This action solved an immediate crisis despite exacerbating the problem that ministers had previously sought to avoid (‘grade inflation’). It produced another year of stark education inequity, but also ensured that inequity went from part of an acute political crisis to its usual status as a chronic low-attention policy problem.

## Introduction

In 2021, the UK government and devolved governments in Northern Ireland, Scotland, and Wales sought to avoid a second successive education policy fiasco. This opportunity related to a second year of COVID-19-related school closures, prompting the cancellation of school exams and search for a feasible alternative. It also followed a crisis in 2020, during which each government had selected two very different policy solutions in quick succession. They chose a standardisation process, based on the argument that teacher assessed grades would undermine the integrity of the exams system by creating unfair results and causing ‘grade inflation’. Then they chose to rely solely on teacher-assessed grades following a public, media, and parliamentary backlash to the unfairness of the standardisation process.

Kippin and Cairney (2021) show that the order of choice, during multiple ‘windows of opportunity’, was crucial to the outcome in 2020. A policy solution that had been *rejected strongly* by each government during the first window (April-June 2020) became a *lifeline* during the second (August 2020). This sequence had a further impact: teacher-assessed grades *remained the only feasible solution in 2021* after the standardisation process became so discredited and no feasible alternative emerged. Each government’s immediate marker of success was to avoid the same U-turn. They still cancelled exams, and sought teacher assessments to determine their grades, but this time without an algorithm to standardise the results. The 2021 process produced *some* of the same concerns about ‘grade inflation’ and the unfair results of a decentralised process, but these issues were no match for the prospect of a second successive political crisis.

Building on Kippin and Cairney’s (2021, p. 3) analysis, we explain these developments by treating the UK and devolved government experiences as ‘variations on the same theme’ to narrate a ‘four-nations’ approach to crisis management and fiasco avoidance during this year’s window of opportunity.

First, we use the same guiding question (2021, p. 2): *what policy solution did each ‘window of opportunity’ produce*? It allows us to provide a systematic comparison between policy choices in 2020 and 2021 by drawing on the same conceptual framework, using:

1. *Multiple streams analysis* to explore this new window of opportunity addressed by four governments. Policymaker attention to the problem was high, few solutions were technically and politically feasible, and each government had to select one quickly.
2. *Heresthetic* to describe the strategic context. Each government’s previous choices influenced their current choices.
3. *Learning and transfer* to identify how the UK and devolved governments coordinated their activities and responded to each other’s choices. As in 2020, there was some focus on a ‘four-nations’ approach, but this time with no gap between exams results days in Scotland and the rest of the UK, and more potential for the governments in Wales and Northern Ireland to avoid following the UK government’s lead.

Second, we focus on how these dynamics relate to the avoidance of policy failure. We draw on studies of policy success and crisis management to explain a ‘four-nations’ approach to avoiding catastrophe. Broadly defined, crises are decision-points characterised by ‘threat’ (such as to the education system, or the government’s legitimacy), ‘urgency’ (prompted by COVID-19 and government choices), and ‘uncertainty’ (about a novel pandemic, and the likely impact of new policies) (Boin et al. 2017, p. 7).

This focus on the need to perform successful policy management during a crisis is crucial to understanding problem definition and the search for solutions. In 2020, each government emphasised one long-term issue (*exam system integrity*) by minimising ‘grade inflation’, rather than another (*equity*) by minimising the ‘achievement gap’ between students in more or less deprived areas. In 2021, their memories of a vivid event were still fresh, prompting them to focus on a short-term issue (*avoiding policy failure*) and accept that they would exacerbate both long-term issues. They focussed on avoiding blame for a crisis-fuelled fiasco (Boin et al. 2009) by using short-term measures of success—will it affect my popularity (*political*) and will it be easy to process and maintain support (*process*)—over a *programmatic* measure of success: will it produce the intended policy outcomes (McConnell 2010)?

Third, we present an empirical account of the latest window of opportunity for each government, guided by the following questions:

- How did each government define this year’s policy problem?
- Which policy solutions were feasible this time?
- What motivated each government to select this year’s solution?

As in 2020, the exams crisis in 2021 suggest that nobody would design this series of choices so badly on purpose, but each choice still has a profound impact on the next. By 2021, governments focussed on narrating a better story of their governing competence during crisis, hoping that their audience would buy it. This short-term success can still occur when governments are failing to deliver long-term promises.

Finally, we reflect on the implications for each government’s vague commitment to education equity. Broadly speaking, to produce greater equity is to reduce unfair inequalities. However, there are two competing grand narratives on what inequalities to address: (1) a ‘neoliberal’ approach emphasises equality of opportunity to access high-quality education, via school or education system performance management; or (2) a ‘social justice’ approach seeks greater equality of outcomes, by addressing ‘out of school’ factors such as poverty and marginalisation (Cairney and Kippin 2021). Each government is somewhat committed rhetorically to the latter story—such as to close the ‘achievement gap’—but has been willing to forego this aim to avoid crises. In 2021, while policymakers made general references to fairness, their attention focussed on addressing short-term issues regarding the opportunity to complete exam-like work rather than longer term trends of unequal attainment. While there was some post-exam-day attention to the unfairness of 2021’s results, the issue of education inequity returned to its usual status as a separate chronic problem, not entwined with an acute political crisis to be solved this year.

## Method and sources

This article uses a theory-informed qualitative method (Vromen 2017). The first half is theory-driven and deductive. We synthesise key insights from policy theories and concepts to create a systematic way to analyse four distinctive policy processes. The second is inductive, drawing extensively from sources in the public record and using documentary analysis to produce a detailed narrative of key choices and events. We draw particularly heavily on news sources such as local and national newspapers, and transcripts of proceedings in forums such as debates in legislatures and committee evidence sessions. These capture not just the (public) rationales used by key policymakers, but also the response and criticism of other relevant policy participants. To prevent an overload of information, we summarise these key sources and choices in the main text and provide a fuller account and exemplar quotations in the Appendix.

## The window of opportunity, sequence, and coordination of policy

Kippin and Cairney (2021, p. 4) describe three ways to connect policy choices in 2020 and 2021. First, multiple streams analysis (MSA) captures a ‘sense of policymaking disorder’ when ‘policymakers responded quickly to events not in their control and produced solutions that did not resolve the problem’. MSA describes a simple contrast between real world versus ‘rational’ and orderly policymaking via a ‘policy cycle’. Both share a focus on the policymaking functions *required* for policy change, including the following: define a policy problem, generate solutions, and select the most feasible solution. However, the policy cycle describes the fulfilment of these functions as orderly and sequential, while Kingdon (1984) describes them as independent streams that only contribute to policy change when they come together during a ‘window of opportunity’:

1. Problem stream: there is high attention to a particular way to define a policy problem.
2. Policy stream: there is already a technically and politically feasible solution to that problem.
3. Politics stream: policymakers have the motive and opportunity to select that solution’ (Kippin and Cairney 2021, p. 4).

In other words, many necessary but insufficient conditions need to be met at the same time to enable policy change (Shephard et al. 2020; Herweg et al. 2018). Policymakers tend to pay attention to few issues, and policy change is not inevitable after a rise in attention. Rather, it may rise and fall before a technically and politically feasible solution emerges and policymakers are motivated to act. Windows of opportunity for policy change may open but close before substantive action takes place.

Second, however, we seek to explain an unusual *series of windows of opportunity* when the absence of policy change was not an option following the COVID-19 pandemic. We need to understand the ‘profound impact of one window on the limits to choice in another’ (Kippin and Cairney 2021, p. 6). The rules of choice matter when policy preferences are intransitive and policymakers have limited resources (such as time and cognition) to help them deliberate. As Riker (1986) describes in relation to heresthetic (the manipulation of choice processes): the sequence of choice, number of options considered (and when), framing of each solution, and intelligibility of each option can help tip the balance from one choice to another.

Third, while all four governments faced similar scenarios—and were committed to learning from each other’s experiences—their willingness and power to go their own way varies (Keating et al. 2012). The UK government led a ‘four-nations’ approach to COVID-19 policy and coordinated key choices such as school closures. When choosing an alternative to exams, UK government policy for England had a disproportionate impact on devolved government policy (Kippin and Cairney 2021, p. 8).

## Narrating success and avoiding policy failure

The vivid memory of policy failure in 2020 put unusually high pressure on each government to find success in 2021. As such, we use conceptual insights on crisis management to help us connect (1) ministerial actions during each window and (2) heresthetic, where there is an unusually restrictive agenda in relation to their available choices, time, and the story to tell about choices. We focus on ministerial perspectives and strategies, but often to show that policy processes are not in their control.

### What does success and failure look like to ministers?

Political science emphasises the contested nature of evaluation, in which actors refer to different goals, values, indicators, evidence, and expectations to declare policy success and failure (Bovens et al. 2001; Boyne 2003; McConnell 2010). Policy success requires ‘craft work’ to produce solutions with a substantive impact on the problem, and ‘political work’ to generate supportive coalitions and maintain legitimacy and support (Compton and ‘t Hart 2019, p. 2–3). In that context, Marsh and McConnell (2010, p. 571) identify three categories to which policymakers may refer when seeking and describing their success:

1. *Political*. Will this policy boost my government’s credibility and chances of re-election?
2. *Process*. Will it be straightforward to legitimise and maintain support for this policy?
3. *Programmatic*. Will it achieve its stated objectives and produce beneficial outcomes if implemented?

While they seek success on all counts, the comparison highlights the trade-offs they make when not all are in their grasp.

### Are ministers in control of their strategic context?

Policymakers may use such questions to seek success, but their powers are not clear. Hay’s (2009) discussion of King Canute helps to demonstrate this point and relate it to UK and devolved government (Cairney and Kippin 2023). Hay (2009, p. 261) explores several tales of Canute: he was *arrogant*, convinced that he could control the sea, *humble*, showing his courtiers that he could not, or he *staged* the event to show the public the limits to his powers (Hay 2009, p. 261).

We can explore similar stories of ministers. First, can they exert control over policy outcomes, and do they think they are in control or try to demonstrate to the public the limits to their influence? Second, if ministers fail to get the policy outcomes they want, does it reflect poor policymaking in specific cases or the general limits to their powers in all cases? It is possible for actors with different worldviews (or political objectives) to produce very different answers to these questions (Hay 2009, p. 263; Bovens et al. 2001, pp. 3–8). Some explanations of policy failure would conclude that ministers had the policy levers at their disposal and were too incompetent to use them. Others would argue that ministers have so many limitations on their resources to address problems—including attention, time, information, cognitive ability, and effective policy instruments—that is misleading to describe ‘levers’ in relation to complex policymaking environments out of policymaker control.

### How do crises affect these dynamics?

Boin et al (2009, pp. 83, 84) relate similar questions to ‘crisis exploitation’, or the use of ‘crisis-type rhetoric to significantly alter levels of political support for public office-holders and public policies’. This contestation to *narrate* crises includes to minimise or maximise their importance; and identify their cause, including who or what to blame or defend (2009, pp. 85–88). In that context, office-holders face the following:

1. *More or less damaging scenarios*.
  • Crises are not damaging when they are absolved of blame.
  • They are potentially damaging when they become the focus of blame and accept responsibility (‘blame acceptance’) or deny responsibility (‘blame showdown’).
2. Multiple strategic choices.
  • They anticipate (a) how their critics will exploit events to undermine them, and (b) the risk/reward of seeking to avoid blame or accept it and mitigate the fallout (2009, p. 89).
  • They consider how much policy change to pursue, including resisting change, and risking its imposition; or, containing it by negotiating an ‘incremental adjustment’ or producing a ‘major and swift rhetorical/ symbolic change’ (2009, p. 90).
3. *A context out of their control*. Their ‘survival’ is boosted if they:
  • ‘Have a good stock of pre-crisis political capital with key media actors’.
  • Narrate their role well (e.g. the cause of the crisis was ‘exogenous’).
  • Have not been in government long.
  • Can use an ‘expert commission’ to investigate what went wrong (2009, p. 100).

### How do ministers portray their actions, and what is the impact on their audience?

These studies show that ministers are key participants in crisis management and *narration*. In Boin et al’s (2017, p. 15–19) terms, they are in the business of ‘strategic crisis leadership’. First they are making sense of a new crisis, such as by drawing on new scientific information. Second, they are taking charge and making hard choices, such as by closing schools and seeking alternative arrangements. Third, they are trying to win ‘framing contests’ by producing an ‘authoritative account of what is going on’, such as by turning a complex problem into the need for exam systems integrity. Fourth, they are fostering a return to a ‘sense of normalcy’ after heightened anxiety, such as by addressing acute, and postponing chronic, problems. Fifth, they are learning how to anticipate future problems and avoid the mistakes of the past, by seeking better solutions and to avoid blame more effectively.

In that context, their ‘political skill and artistry’ influences the order and presentation of choices during crisis (Riker 1980, p. 445; Hay 2009, pp. 276–277). Their failure-avoidance relates to (1) choosing which criteria to evaluate success, *and* (2) their stories of their governing competence. However, their choices and stories are limited by their environments and audiences. In Westminster systems, they may seek pragmatic ways to respond to dynamics out of their control, but are expected to be in charge and accountable for government policy (Cairney 2020a). They act strategically to make and narrate choices, but face competition from their critics, and do not control the reaction of their audiences (Ball et al. 2021; Cairney 2020b, pp. 66–68; Boin et al. 2020).

## The latest window of opportunity: dealing with past failure

These factors—the sequence of windows, need to narrate crisis management, and four nations dynamic—explain the key choices of 2020 and context for 2021.

*Policy window 1* (March-August 2020). The closure of schools in March 2020 prompted the first window of opportunity for major—but temporary—policy change during a public health crisis: the school exams replacement policy. Policymakers only had weeks to design and select a replacement. Each government narrated its crisis management in similar ways, emphasising the need for *policy continuity* (to maintain the integrity of exam result outcomes) and *stability* (to protect the recruitment of students to further and higher education). Their definition favoured only two feasible solutions: (1) to use teacher assessed grades alone or (2) standardised (using an algorithm) to reduce unfair variations and minimise grade inflation. Each government was motivated to select and defend the latter, even during a crisis when public, media, and parliamentary concern was high.

*Policy window 2* (August 2020). The second solution became necessary when the first failed. Policymakers had days to decide to defend option 1 or revert to option 2 (there was no option 3). Then, they abandoned both crisis management narratives—(1) this is the best solution, and (2) we can address your concerns about its impact—within days. The Scottish and UK Governments accepted blame for the initial choice (and, to some extent, their handling of the outcry). This need to address a dual crisis—regarding the problem and their management—prompted a major policy and narrative reversal: the solution that they had rejected wholeheartedly became a lifeline and the only feasible alternative. The Welsh and Northern Ireland governments told similar stories, but with more blame placed on the UK government and the spill-over effect of its policy reversal.

*Policy window 3* (October 2020 onwards). A third solution was required for 2021 as soon as all four governments announced a second year without school exams (see Annex Table 1 on each government’s timing). Policymakers had more time and experience, but the failure of their first solution, and selection of a second, became the core reference point.

While the experience of each government varied *somewhat* in 2020, they all faced blurry boundaries between the ‘problem’, ‘policy’, and ‘politics’ streams. Each government’s policy problem was *ostensibly* to find a feasible alternative to school exams, but *actually* to avoid a cumulative sense of political failure (2021, pp. 14, 15). The motivation of policymakers was to select a solution to a policy problem (how to assign grades in the absence of exams) and political problem (how to avoid exacerbating or repeating a crisis) (2021, pp. 15, 16). This experience of 2020 provides the context for 2021, providing further need to understand the sequence and coordination of choice during multiple windows of opportunity.

## How did each government define the problem this time?

All four governments initially sought to hold in-person exams in 2021 before admitting defeat (and facing criticism about delaying the inevitable). The timing of their choices varied markedly. However, upon cancellation, their definition of the problem was similar, containing three requirements:

1. to assign ‘fair’ grades in the absence of exams (although a clear definition of fairness is hard to find),
2. to avoid the use of an algorithm to standardise teacher-assessed grades (to avoid a political outcry), *but also*
3. to deliver consistent grades at a national level from local examinations centres (to take a less top-down approach to top-down uniformity).

Initially, their aim was to mitigate the negative effects of the pandemic on school attendance and any shift to online learning. They each focussed on how to modify the timing, difficulty, and conditions of exams to ensure they go ahead in ways that made assessment less burdensome to students (Vesty et al. 2020; Morris 2021; UK Government, 2020; Weir 2021a). In 2020, a ‘four-nations’ approach had produced a coordinated timing of cancellation. For 2021, they announced at different times (Annex Table 1), with Scotland and Wales leading the way between October and December 2020, and the UK and Northern Ireland doing so only following the imposition of new lockdown measures in January 2021.

As in 2020, the problem was how to provide an alternative to school exams. However, this earlier cancellation (and previous knowledge that cancellation may be inevitable) gave each government more time to plan. Further, their 2020 experience gave them more knowledge of the political feasibility of their next moves. This knowledge is reflected in each government’s discussion of planning and next steps, signalling a desire to not repeat the fiasco associated with algorithms to standardise exam outcomes.

The Scottish Government commissioned Professor Mark Priestley (August 2020) to ‘lead an independent review of the processes through which National Qualifications were awarded in 2020 after exams were cancelled due to the coronavirus pandemic’ (Priestley, 2020). Swinney used the recommendations to justify the replacement system for Standard 5 examinations on 7^th^ October 2020. He stated: ‘there will be no algorithm’ (Wearmouth 2021). He sought a different way to mitigate the risk of the differential treatment of students in different examinations centres in the absence of national standardisation (Scottish Parliament Official Report 7 October 2020, col 50, 51). As such, the Scottish Government defined the problem as the need to apportion grades in the absence of exams, in a manner which avoided altering individual student grades routinely during a standardisation process, and which delivered consistent grades across centres. Further, teachers must be the key figures in deciding those grades. When Higher and Advanced Higher exams were cancelled, this same requirement applied to these awards.

The Welsh Government made similar moves and statements, with Kirsty Williams justifying exam cancellation with reference to ‘the wellbeing of learners and fairness across the system’ (BBC 2020b), then seeking consistency across different examinations centres (Welsh Government 2020a). The stated (if vague) desire for ‘fairness’ was reflected in the priorities of influential bodies such as the Independent Review (akin to the Priestley report) and Qualification Wales (which had publicly urged cancelling exams) (BBC 2020c; Welsh Government 2020b). Although they did not define fairness, we can observe (1) a rejection of last year’s algorithmic approach, *but also* (2) the search for consistency across different centres.

The UK Government told a similar story of the policy problem. Gavin Williamson told the House of Commons that ‘this year, we will put our trust in teachers rather than algorithms’ while describing the importance of ‘fairness and consistency’ in allocating grades (HC Deb, 6 January 2021). The latter related partly to the patchy and unequal access to education during COVID-19 restrictions, requiring ‘a system that would accommodate the very different learning experiences that students had had’ and allow a student to achieve the same grade as a student with the same performance in a different part of England and a previous year (Simon Lebus, Chair of Ofqual, to the House of Commons Education Committee 2021).

The problem framing in Northern Ireland was *mostly* similar. Peter Weir proposed an alternative to examinations that would ‘ensure fairness and consistency, without ‘statistical standardisation using an algorithm’ (Meredith, 2020). One significant difference is the presence of a distinctive exams element in Northern Ireland: the ‘transfer tests’ used to determine post-primary school entry into (selective) grammar schools. They were initially postponed and then later cancelled by the AQE and PPTC administrative bodies in January 2021, adding pressure to Weir and highlighting debates on the test’s unequal impact (Deeney 2021; Weir 2021b). Weir framed the cancellation of GCSEs and A-Levels in terms of his unwillingness to countenance Northern Irish students being disadvantaged relative to their other UK counterparts (Press Association, 2021; Northern Ireland Department of Education, 2021). While Weir was the only Minister to say so publicly, a ‘four-nations’ approach is important to the devolved governments seeking to address spill-over effects from the UK Government.

## Which solutions were feasible this time?

To all intents and purposes, two solutions were deemed unfeasible or, at least, not worth considering in their ‘pure’ form:

1. Any version of 2020s solution, to use a mathematical model to ensure algorithmic standardisation. The idea of ‘an algorithm’ was rejected explicitly by three of the four education ministers (and implicitly by Williams). In theory, a modified and more equitable solution of this kind could have been proposed (Everett [Bibr CR20][Bibr CR21]). However, after August 2020, the word ‘algorithm’ became an empty vessel which ministers could fill with blame for the fiasco.
2. The rejected solution in 2020, to use unstandardised teacher-assessed grades, even though it became a ‘lifeline’ to ministers in August 2020 (Kippin and Cairney 2021). The ‘pure’ form of this solution was rejected with reference (again) to the potential for (a) inconsistency to reduce faith in individual student results and (b) grade inflation to undermine the credibility of examinations systems.

Instead, ministers sought to describe modifying teacher grades to ensure consistency (in the absence of an algorithm).

Each government commissioned their own reports and consultations to inform policy design. For example, in Scotland, the Priestley report (2020, p. 5) recommended qualifications ‘be awarded on the basis of centre estimation based upon validated assessments’, and ‘the development of a nationally recognised […] system for moderation of centre-based assessment’. Qualifications Wales, Ofqual, and the CCEA in Northern Ireland were each asked to make recommendations for future assessments (despite the bravado of the UK and Northern Irish governments regarding the likelihood of exams going ahead in 2021) (Welsh Government 2020b; Qualifications Wales 2020, Letter to Kirsty Williams, 16 October). Williamson, G., 2021, Letter to Simon Lebus, 13th January; Weir 2021c, Letter to Northern Ireland school principals, 8 January).

However, their conclusions had certain features in common. First, they described a strong role for teacher judgement as a proxy for avoiding the political infeasibility of excessive national standardisation (‘teachers … not algorithms’ – Williamson, HC Deb, 6 January 2021, c763). Second, to foster credibility and national consistency in the absence of an algorithm, they commissioned externally produced assessment materials to be available to schools (for instance, see Welsh Government 2020c). Third, each government created a system for quality assurance at school level (rather than at exam board or exam regulator level), accompanied by the expectation that schools produce clear and substantial evidence of student achievement (Annex Table 2):

- The Scottish Government adopted the ‘Alternative Certification Model’, described by the SQA as results ‘decided by teachers and lecturers using assessments completed by learners that followed the national standard set by SQA’ (Robertson 2021).
- The Welsh Government (2020b) described a ‘three pillar system’ represented by ‘non-examination assessments, internal assessments, and assessments that are externally set and marked’.
- In England, ‘Teacher Assessed Grades’ were approved by heads of department and Head Teachers, then submitted to the exam board. The evidence for grades included mocks, tests, and coursework already been completed, and questions provided by exam boards (Ofqual 2021a).
- In Northern Ireland, the Alternative Awarding Arrangements would see schools present the CCEA with a ‘portfolio of evidence’ including coursework and exam-like materials which Weir stated ‘should not be treated as exams’ (Meredith 2021). The CCEA would have the authority to instruct schools to re-run their process if it found evidence that it had been handled incorrectly.

In other words, each governments favoured a solution that emphasised both key elements:

1. Political feasibility, aided by a rhetorical focus on teacher judgement and school autonomy.
2. Technical feasibility, aided by ‘exam like’ materials provided by exam boards, with quality assurance carried out within and between centres (and a fee going to exams regulators despite the extra burden being placed on schools). The latter highlighted a direct link between the work carried out by students and the grades they received (unlike in 2020 when grades were hypothesised).

This combination of elements allowed each government to propose a feasible-enough solution, in terms of manageable opposition (in the shadow of last year’s fiasco) rather than widespread support. Teachers and students complained about the creation of a shadow exams system (‘exams in all but name’ was a common phrase) and a high workload to demonstrate evidence of achieving each grade, which schools appeared to adopt as the safest measure to meet requirements (Annex Table 1). For example:

- In Scotland, Labour MSP Michael Marra wrote to Swinney, lamenting ‘crammed exam diets that young people are being forced through, despite repeated assurances from yourself and other ministers that there are no exams’ (McCall 2021).
- In Wales, Ruth Davies, President of the school leaders’˙ union NAHT, described ‘a real concern that we will end up with exams by stealth’ (Davies 2020; Gwenllion 2021).
- In England, Ofqual board member Sir John Coles allegedly resigned over the decision to introduce non-compulsory ‘mini exams’ by ‘the back door’ (Winter 2021).
- The Northern Ireland’s Children’s Commissioner Koulla Yiasouma criticised the ‘overuse of controlled assessments in test conditions’ (Colhoun 2021).

However, much like in 2020, there was little evidence of sustained and organised opposition, with unions and other education policy stakeholders offering qualified support for the system or focussing on secondary issues such as timing (in England and Northern Ireland). This opposition was diluted somewhat by complaints about forthcoming grade inflation (particularly in England, where the Chair of the Education Committee Robert Halfon described an ‘all must have prizes’ approach) (Schools North East 2021).

## What motivated policymakers to adopt this choice?

From this experience we can identify five common motivational elements (albeit with variations across the UK). First, to get the timing right, by waiting as long as practicable to maintain hopes for a return to exams, then pivoting when these hopes are dashed. The Welsh and Scottish governments were the first to cancel, producing a longer lead-in to propose and refine their alternative arrangements. The UK Government cancelled later, drawing upon contingency arrangements.

Second, to honour a ‘four-nations’ approach, partly to avoid spill-over effects from each other’s choices (although ‘standardisation’ related to grading within each system only). They did so in a looser way than in 2020, which had exhibited close alignment in the nature and sequencing. This time, each unilateral choice (to respond more to domestic debates) put pressure on the others. In particular, the Northern Ireland Executive sought to not be the odd-one-out if it would have a disproportionately negative effect on its students. Weir criticised Wales’ ‘unilateral’ cancellation of GCSE and A-Level exams and the (more significant) UK Government choice (The Newsroom 2020; Hazell 2021).

Third, to learn from past political mistakes, in the knowledge that there would be high public, media, and parliamentary attention to exam results. This time, they relied more on the credibility afforded by teacher judgement, and shied away from any risk that standardisation would involve the same negative political consequences. For example, Simon Lebus, Chair of Ofqual, described a hypothetical example to demonstrate that their focus would not be to change grades uniformly. Rather, they would use (1) indicators to identify which centres ‘should be looked at in more detail’, and (2) a ‘random sampling process’ to monitor the overall system (House of Commons Education Committee 2021).

Fourth, to act according to feedback from reviews of the previous year (including independent reviews in Scotland and Wales). Further, given that key sources of commissioned reviews (the SQA, Qualifications Wales, Ofqual, and the CCEA) were implicated heavily in the original fiasco, they had a strong incentive to develop proposals to restore their reputations.

Fifth, to address the issues that motivated their choices in 2020, including to maintain the ‘credibility’ of its exams (in relation to avoiding grade inflation) and ensure a smooth transition of students from high school to further and higher education (Kippin and Cairney 2021).

Consequently, while each government planned its own journey, the four governments all responded to multiple (and often contradictory) motivations, and ultimately ended up in a similar place. They selected teacher-assessed grades, underpinned by quality assurance (but not standardisation) carried out at the national level, and ‘exams in all but name’ administered by schools. The latter involved producing evidence of student attainment, collated by teachers, and overseen (but not subject to alteration) by national bodies. In 2020, policymakers were motivated initially to prioritise ‘credibility’ by avoiding grade inflation, then they pivoted quickly to prioritise ‘fairness’ to address the impact of their standardisation algorithm. In 2020, they sought a combination of ‘fairness’ and ‘credibility’, accepting that teacher discretion could help deliver both (if using ‘exam-like’ assessments), and that some grade inflation was a worthwhile price to avoid another fiasco.

In this context, ‘fairness’ refers, remarkably narrowly, to *avoiding the political fallout of the results criticised as unfair in 2020* rather than a more meaningful engagement with their commitments to education equity. We struggled to find any ministerial reflections on the meaning of fairness in relation to ‘social justice’ approaches or long-term trends in unequal exams outcomes (see Cairney and Kippin 2021). Indeed, their focus on handing greater responsibility to others—including teachers and schools—allowed them to limit their role primarily to ensuring an equality of process by monitoring standardisation measures.

## The window of opportunity to avoid a second fiasco

All four governments are responsible for their own education systems and could go their own way. However, they adopted a ‘four-nations’ approach in 2020 and sought *to some extent* to use this model again. More importantly, their approaches in 2020, combined with a further year of COVID-19-related school disruption, limited their options for 2021. Their primary motivation—to avoid a second policy fiasco—informed their definition of the policy problem and the political feasibility of each solution. Their windows of opportunity involved three elements coming together at the same time, in a period when inaction was not an option:

- *Problems*. Their attention rose to a problem defined as (1) finding an alternative to exams following school disruptions, *and* (2) avoiding the same political fallout as last year.
- *Policies*. They ruled out algorithmic standardisation as politically infeasible (the word ‘algorithm’ was toxic, used only to distance ministers from the past) and pure teacher-assessed grades as technically infeasible (without some ways to ensure standardisation). The only feasible option was a system driven by teacher grading, to ensure credibility and a sense of personal attention to quality and fairness, *and* quality assurance, to ensure some standardisation.
- *Politics*. The *opportunity* to act was a given. The *motive* to act was primarily political (to avoid a second fiasco), coupled with a desire to coordinate responses across the UK, learn from past mistakes, incorporate feedback from reviews, *and* address the issues that informed their initial choice in 2020 (to prioritise exam system credibility).

The result was the selection of a solution that reflected mixed motivations. To foster political feasibility, they emphasised the teacher allocation of grades (while knowing that this emphasis was not supported universally). To foster their interpretation of technical feasibility, they fostered ‘exams in all but name’, with quality control carried out within parameters set by exam boards and regulators, and grades generated by bespoke assessments within schools. This solution pleased no-one but, crucially, did not receive enough opposition to force a second U-turn.

Each government’s policies represented variations on the same theme, regarding the timing and emphasis placed upon similar decisions. From mid-October 2020 to January 2021 it seemed possible to expect different policies in Scotland and Wales versus England and Northern Ireland. However, they would all end up pursuing similar policies, in response to (1) public health imperatives and their unequal impact on learning, and (2) the events of 2020 that limited the availability of politically and technically feasible options.

## The aftermath: was it a window of opportunity for equity?

In August 2020, the immediate outcry prompted an accelerated process of reflection and action to address inequitable exam results (unfairly unequal outcomes). While Swinney and Weir faced pressure to resign (Carrell 2020; McNeilly 2020), and Williams received strong criticism (BBC 2020c), Williamson faced probably the most intense pressure, with one poll reporting that 92% of 6000 surveyed teachers agreed that he should resign (Forrest, 2020).

This outcry was not such a feature of August 2021, *partly* because Williamson already seemed to be on borrowed time (lasting until September 2021, then being knighted six months later) and the three devolved education ministers had already been replaced (there were cabinet reshuffles following devolved elections in Scotland and Wales in May 2021 and following a political crisis in Northern Ireland when Paul Givan replaced Arlene Foster as First Minister in June 2021). This absence of equivalent criticism is notable because the immediately available data still highlight—in each country—two kinds of results that policymakers and their critics warned against. First, Table 1 shows that each system produced in 2020 and consolidated in 2021 the ‘grade inflation’ that each government wanted to minimise (before accepting it as part of a lifeline in 2020 and necessary compromise in 2021):

**Table 1:** Selected exam results in each education system, 2019–2021

Students in Scotland achieving:	2019	2020	2021
A/A* National 5	35.1%	42.3%	46.7%
A-C National 5	78.2%	89.0%	85.5%
A/A* Higher	28.3%	40.0%	47.6%
A/A* Advanced Higher	31.8%	46.3%	51.0%
A-C Higher	74.8%	89.3%	87.3%
A-C Advanced Higher	79.4%	93.1%	90.2%
Students in Wales achieving:	2019	2020	2021
A/A* GCSE	18.4%	25.5%	28.7%
A-C GCSE	62.8%	73.8%	73.6%
A/A* A-Level	27.0%	41.8%	48.3%
A-C A-Level	76.3%	91.8%	89.2%
Students in England achieving:	2019	2020	2021
7/A GCSE	20.6%	25.9%	28.5%
4/C GCSE	67.0%	75.9%	76.9%
A/A* A-Level	25.2%	38.1%	44.3%
A-C A-Level	75.5%	87.5%	88.2%
Students in Northern Ireland achieving:	2019	2020	2021
A/A* GCSE	31.4%	37.0%	40.5%
A-C GCSE	81.7%	89.4%	89.3%
A/A* A-Level	30.9%	43.3%	50.8%
A-C A-Level	84.8%	95.0%	94.6%

*Sources* JCQ (2019a, 2019b, 2020, 2021), SQA (2021)

Second, Table 2 shows that the 2021 results provide further evidence of a widening or steady ‘attainment gap’ between state versus private schools. Each country’s results provoked *some* criticism of the continuation or deepening of the ‘attainment gap’ within state schools and in relation to private schools (Holden 2021 on Scotland; Wightwick 2021 on Wales; Harris 2021 on Northern Ireland), while the Chair of the House of Commons Education Select Committee describing private school students in England as having ‘[…] a superfast lift at the top of the skyscraper, and the state schools have been on a fast escalator’ (House of Commons Education Committee 2021b). There is also evidence of longstanding inequalities *within* all four state school systems which have either persisted or widened during the past three years, which can be seen in a comparison of the allocation of top grades:

- There was a 25.1 percentage point gap between students in Scotland’s least and most deprived areas in achieving an A at their National 5 exams in 2021 (the figure was 27.6 in 2019) (SQA 2021b).
- In Wales, there was a 21.1% gap between students eligible and ineligible for free school meals attaining an A or A* grade (in 2019, the figure was 14.1) (Qualifications Wales 2021).
- In England, there was a 17 percentage point gap between pupils eligible for free school meals and those ineligible in receiving grades 7 and above (an increase of 2.27 percentage points since 2019) (Lee 2021).
- In Northern Ireland, there was a 34 percentage point gap between pupils achieving an A or above who attended a grammar school vs those who attended a non-selective school (CCEA 2021).

However, a combination of (1) quality assurance measures focussing on evidence for attainment, and (2) the lack of algorithmic downgrading, created sufficient legitimacy for the new approaches. Or, at least, there was no equivalent focal point for campaigning against inequalities even though they still exist. In 2021, educational inequality moved from (1) an acute crisis to be addressed in a matter of days to (2) a longstanding ‘chronic’ problem to be placed in the ‘too hard’ pile.

**Table 2:** Qualifications by centre type 2019–2021 in Scotland, Wales, England, and Northern Ireland

	State Schools and colleges			Independent Schools*		
Students in Scotland achieving:	2019	2020	2021	2019	2020	2021
A/A* National 5	32.9%	40.4%	44.8%	68.9%	72.9%	76.9%
A-C National 5	77.2%	88.5%	85.1%	94.8%	97.7%	96.8%
A/A* Higher	25.7%	37.7%	45.4%	58.1%	67.0%	75.6%
A/A* Advanced Higher	27.3%	41.7%	46.7%	53.3%	66.8%	72.3%
A-C Higher	73.2%	88.6%	86.5%	92.8%	97.7%	97.2%
A-C Advanced Higher	76.9%	92.0%	88.1%	91.5%	97.9%	96.9%
Students in Wales achieving:	2019	2020	2021	2019	2020	2021
A/A* GCSE	18.7%	25.9%	29.3%	53.7%	61.6%	66.5%
A*-C GCSE	64.4%	74.8%	74.6%	88.1%	95.4%	95.2%
A/A* A-Level	24.6%	40.6%	46.0%	68.2%	80.5%	85.2%
A-C A-Level	76.1%	92.2%	88.9%	93.3%	98.9%	98.3%
Students in England achieving:	2019	2020	2021	2019	2020	2021
A/A* GCSE (equivalent)	19.8%	25.02%	27.3%	46.6%	57.2%	61.2%
A-C GCSE (equivalent)	68.4%	77.4%	77.7%	89.9%	95.6%	95.5%
A/A* A-Level	22.1%	34.0%	39.3%	44.0%	60.8%	70.1%
A-C A-Level	75.4%	86.0%	86.6%	87.5%	95.8%	96.5%
Students in Northern Ireland achieving:	2019	2020	2021	2019	2020	2021
A/A* GCSE	12.7%	18.0%	22.2%	47.6%	53.8%	56.5%
A-C GCSE	70.1%	81.5%	81.4%	94.4%	97.6%	97.3%
A/A* A-Level	13.7%	25.2%	36.6%	38.5%	52.1%	57.9%
A-C A-Level	73.7%	90.5%	90.4%	91.2%	97.6%	96.6%

*Sources* author’s own, adapted from available data in SQA (2021a), Qualifications Wales (2021), JCQ (2021), Ofqual (2021b), JCQ (2021, 2020), CCEA (2021), JCQ (2021, 2020)

Note: Northern Ireland figures are for Grammar Schools not independent schools

In other words, the cancellation of exams in 2021 opened a window of opportunity for a policy solution designed to (1) allow ministers to avoid the most egregious and high salience examples of unequal outcomes and (2) control the political damage to their government. It did not provide a window to actually improve education equity. The latter may require a more fundamental challenge to the dominant paradigm contributing to inequitable outcomes. Instead, these actions took place within the ‘neoliberal’ paradigm dominant across the UK (albeit with Scottish and Welsh variants emphasising distance from the UK government’s harshest edges). This approach fosters a narrow and instrumental definition of equity—to prioritise the mythical idea of equal access to high-quality schooling—as part of an economic definition of education, emphasising its contribution to a global knowledge economy, and encouraging (1) international competition between education systems and (2) domestic competition between schools within each system. It is this approach that each government’s policy solutions sought to protect when prioritising their ‘credibility’.

In other words, although we witnessed unusual attention to ‘fairness’ in 2020 and 2021, there was no motivation to exploit a window of opportunity for an alternative ‘social justice’ paradigm, such as by (1) emphasising education’s emancipatory value, (2) fostering an inclusive definition of equity based on ‘out of school’ factors (such as poverty), (3) emphasising the need to foster each student’s ‘capabilities’, or (4) addressing marginalisation, such as by challenging racial discrimination or providing unequal resources to foster more equal attainment outcomes (see Cairney and Kippin 2021 for a review of neoliberal versus social justice debates in education research).

## Conclusion

All four governments of the UK each produced three exams replacement policy solutions in little over a year: (1) introducing standardised teacher assessed grades aided by their notorious algorithms, (2) removing standardisation within days of the exam results in 2020, and (3) using teacher-assessed grades backed by exam-like assessments to generate evidence of attainment. Kippin and Cairney (2021, p. 18) describe the second solution as a rapid ‘U turn’ and the combination of 1 and 2 as a ‘fiasco’ because ‘Had they selected this policy in the beginning, they could have invested more resources in its planning, delivery, and consistency’ (2021, p. 18). They had this time for 2021, but held on to the idea of returning to exams for months (2–3 in Wales and Scotland; almost 5 in England and Northern Ireland) and generated a similar sense of haphazard crisis management.

The timing and sequence of these choices matter as much as their substance. The first policy solution had to be found as soon as school exams were cancelled in March 2020. Policymakers only had *weeks* to design and select a replacement. The second solution became necessary when the first one failed immediately in August 2020. Policymakers had *days* to decide to defend their choice or revert to plan B (there was no plan C). A third solution was required for 2021 as soon as all four governments announced a second year without traditional school exams. Policymakers had far more time and experience, but the failure of their first solution—and selection of a second—became the key reference point: a solution *rejected wholeheartedly* before August 2020 became a *lifeline* after the exams fiasco then the basis for a third solution’s political feasibility.

Our analysis of each government’s reasoning shows that *this third solution is not what each government would have selected for 2021 in the absence of the sequence of events in 2020*. Each minister faced immense pressure to avoid the failure of the previous year, and this constraint informed every aspect of their windows of opportunity, including how they defined the policy problem and assessed the feasibility of each solution. They sought to assign ‘fair’ grades in the absence of exams (without providing a clear definition of fairness); avoid any algorithm to standardise teacher-assessed grades (and therefore avoid another political outcry); *and* deliver consistent and credible grades at a national level *but* without interfering too much in local examinations centres and school decisions. As in 2020, they selected what seemed to be the most politically feasible option even though it suffered from major technical problems (and the burden on students and teachers went beyond technical concerns).

This focus on timing and sequence should not be overshadowed by a focus on specific policy solutions. Describing ‘why the standardisation process and algorithm went so wrong’ without describing ‘why the policymaking process went so wrong’ only tells part of the story (Kippin and Cairney 2021, p. 19). Further, neither should overshadow the absence of educational equity during this period. In 2020, each government could not have done a better job, of highlighting the unfair and enduring inequalities of attainment across the UK, if they tried. In 2021, they each focussed on quality assurance to avoid another fiasco that would undermine the credibility of their exams systems. In doing so, they reasserted a neoliberal approach to education, downplayed social justice, and succeeded in putting education inequity back on the ‘too hard’ pile.
